# Acoustic wave in a suspension of magnetic nanoparticle with sodium oleate coating

**DOI:** 10.1007/s11051-014-2271-z

**Published:** 2014-02-05

**Authors:** A. Józefczak, T. Hornowski, V. Závišová, A. Skumiel, M. Kubovčíková, M. Timko

**Affiliations:** 1 Institute of Acoustics, Faculty of Physics, Adam Mickiewicz University, Umultowska 85, 61-614 Poznan, Poland; 2 Institute of Experimental Physics, Slovak Academy of Sciences, Watsonova 47, 040 01 Kosice, Slovakia

**Keywords:** Nanoparticles, Magnetic fluid, Velocity and absorption of ultrasound, Hydrodynamic size

## Abstract

The ultrasonic propagation in the water-based magnetic fluid with doubled layered surfactant shell was studied. The measurements were carried out both in the presence as well as in the absence of the external magnetic field. The thickness of the surfactant shell was evaluated by comparing the mean size of magnetic grain extracted from magnetization curve with the mean hydrodynamic diameter obtained from differential centrifugal sedimentation method. The thickness of surfactant shell was used to estimate volume fraction of the particle aggregates consisted of magnetite grain and surfactant layer. From the ultrasonic velocity measurements in the absence of the applied magnetic field, the adiabatic compressibility of the particle aggregates was determined. In the external magnetic field, the magnetic fluid studied in this article becomes acoustically anisotropic, i.e., velocity and attenuation of the ultrasonic wave depend on the angle between the wave vector and the direction of the magnetic field. The results of the ultrasonic measurements in the external magnetic field were compared with the hydrodynamic theory of Ovchinnikov and Sokolov (velocity) and with the internal chain dynamics model of Shliomis, Mond and Morozov (attenuation).

## Introduction

Magnetic nanoparticles have been studied extensively in recent years because they have a vast potential for application in many different areas of biomedicine, from diagnostics to treatment of diseases. For medical applications nanoparticles require highly biocompatible particle surfaces. Biogenic magnetoparticles such as bacterial magnetosome particles, derived from various magnetotactic bacteria such as *Magnetospirillum magnetotacticum*, are organelles consisting of magnetite crystals enclosed by a phospholipid membrane that offers a high degree of biocompatibility (Han et al. 2007; Timko et al. 2009). For chemically obtained particles, chemical modification of the nanoparticles surface is necessary. Many synthetic and natural polymers such as dextran, polyethylene glycol (PEG), or chitosan are biocompatible and may be used as coatings. Sodium oleate may also provide a biocompatible layer. As an amphiphilic surfactant, it has much higher affinity to nanoparticle surfaces compared to other surfactants (Jiang et al. 2010). Sun et al. (2007) showed that sodium oleate used as a coating for magnetite nanoparticles leads to a lower toxicity and better magnetic properties than PEG. The saturation magnetization of the magnetite nanoparticles coated with sodium oleate was greater than that of magnetite nanoparticles of the same size coated with PEG.

The delivery of nanoparticles into a human body usually requires suspending the nanoparticles in a water-based fluid. Such colloidal suspension of monodomain magnetic particles is called magnetic fluid (nanofluid) or ferrofluid and has properties between a conventional, isotropic liquid without the presence of a magnetic field and solid-like medium after its applying. When a magnetic fluid is submitted to the external magnetic field, the formation of internal structure is observed depending on the initial volume concentration, and on the coupling constant, *λ* = *μ*_0_ *πM*_b_^2^ *d* ^3^/144*k*_B_ *T*, that measures the magnetic attraction of magnetic particles in a given temperature. In the equation for the coupling constant, *μ*_0_ = 4*π* × 10^−7^ H m^−1^ is the magnetic permeability of vacuum, *M*_b_ is the bulk magnetization of the particle material, and *d* is the diameter of the particle. Bigger magnetic particles (with diameter above 16 nm) have higher dipolar strength and associate in flexible chain aggregates, the number and length of which increase with the magnetic particles concentration and with the strength of an external magnetic field (Mendelev and Ivanov 2004; Morozov and Shliomis 2004). Although mean magnetic diameter for typical magnetic fluid is about 10 nm and magnetic dipolar interaction between them is too weak to provide their aggregation into flexible chains, the real magnetic fluids are always polydisperse and the magnetic interaction between the biggest particles, corresponding to the tails of the particle size distribution (PSD), is strong enough for the magnetic nanoparticles to form the internal structures.

In this work, the propagation of acoustic wave in the magnetic fluid stabilized by two-layer shell is described. Interest in the interaction of the acoustic waves with colloidal media has a long history (Challis et al. 2005). The application of the ultrasound techniques to the study of the properties of magnetic fluids started in the late 1970s with the pioneering work of Chung and Isler (1978) and continuing until today (Skumiel et al. 2003; Józefczak and Skumiel 2006; Charaziak et al. 2008; Hornowski et al. 2008; Motozawa et al. 2008; Rozynek et al. 2011; Rashin and Hemalatha 2012; Kúdelčík et al. 2013). The description of magnetic fluids by ultrasound requires formal theoretical basis which relates the properties of the medium to the complex wavenumber governing propagation. In the absence of a magnetic field, magnetic fluid can be described as a suspension of solid magnetic particles stabilized with surfactant layer of nanometer size. There is an abundance of theoretical models on acoustic wave propagation in particulate mixtures starting from the early works of Urick (1947, 1948; Urick and Ament 1949) to the scattering models of Epstein and Carhart (1953), and others (Challis et al. 2005). However, the applying of external magnetic field makes the theory of ultrasonic propagation in a magnetic fluid far more complicated. Such theory must account for the dependency of ultrasonic velocity and attenuation on magnetic field strength, frequency, and the angle between magnetic field and the propagation direction of the acoustic wave (ultrasonic anisotropy). The theoretical models of propagation of acoustic waves in magnetic fluids evolved over the last thirty years, and today they fall into two broad categories: hydrodynamic—both one-phase (Parsons 1975; Gotoh and Chung 1984; Müller et al. 2003; Ovchinnikov and Sokolov 2009, 2013), or two-phase (Kaczmarek et al. 2000; Hornowski et al. 2010)—and models based on the internal chain dynamics (Taketomi 1986; Pleiner and Brand 1990; Shliomis et al. 2008). The ultrasonic techniques are especially useful for detecting small deviation of ferrofluid features from ideality due to the internal chain formation. The ideality means that magnetic particles interact with external magnetic field but do not interact with each other.

In this article, the various methods—magnetic, microscopic, based on the scattering of light, rheological, and ultrasonic—were used to study properties of the magnetic fluid stabilized with interdigitated bilayer. A special care was taken in combining the results from different methods in such a way that leads to the coherent description of ultrasonic results with a minimal number of fitted physical parameters. The ultrasonic velocity in the absence of the magnetic field was analyzed within the framework of the modified model of Urick (Pinfield et al. 1995) describing sound propagation in particulate medium—a model which allows one to calculate the adiabatic compressibility and its temperature variations of the magnetic core-surfactant layers aggregate. The ultrasonic experimental results in the external magnetic field were described using two theoretical approaches: for the analysis of velocity data, the hydrodynamic model of Ovchinnikov and Sokolov was used (Ovchinnikov and Sokolov 2009, 2013), whereas for attenuation resulted from the internal chain dynamics the theory of Shliomis et al. (2008) was adopted.

## Sample preparation

**Fig. 1:**
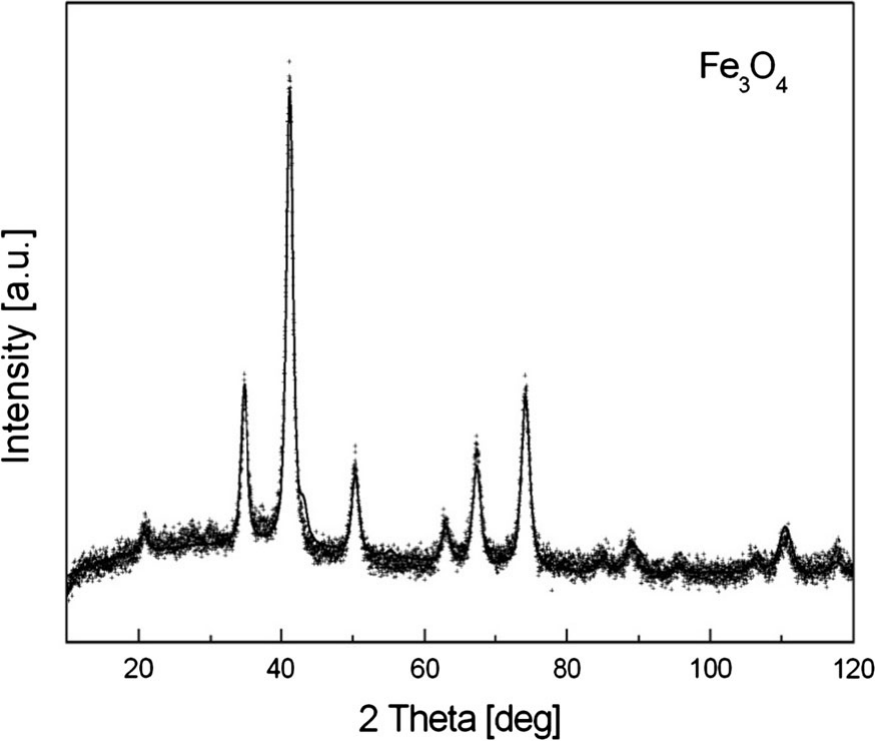
XRD spectrum of prepared magnetite particles

**Fig. 2:**
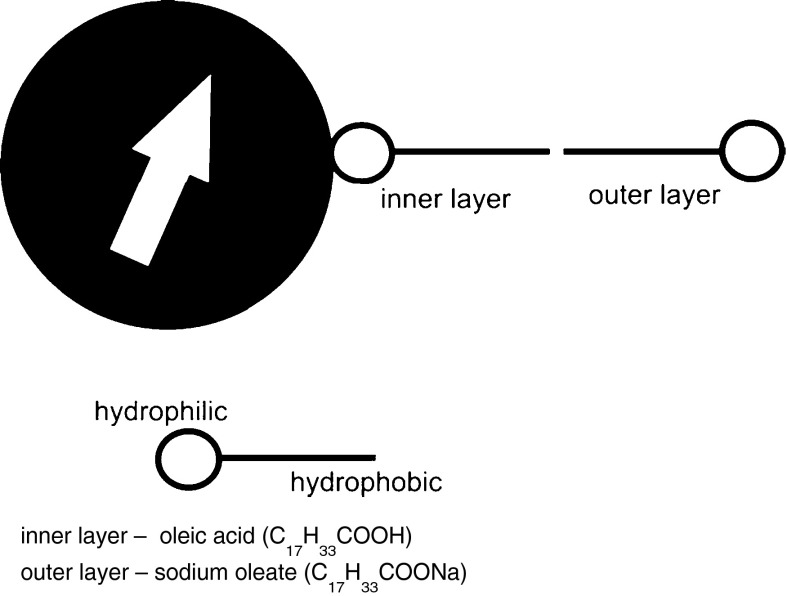
The formation of magnetic particle with interdigitated bilayer

The nanoparticles have core–shells structure. The co-precipitation method of ferric and ferrous salts in an alkaline aqueous medium was used to prepare spherical magnetite particles. In a typical synthesis, aqueous solution of 2.9 g FeCl_3_ × 6H_2_O and 1.5 g FeSO_4_ × 7H_2_O in a molar ratio 2:1 was prepared by dissolving in deionized water. To this mixture, an excess of hydroxide ions (8 ml NH_4_OH) was added at room temperature under vigorous stirring. Black precipitate of magnetite nanoparticles was immediately formed. X-ray diffraction measurement was performed to identify the crystallographic structure of prepared iron oxide particles. The XRD spectrum of the prepared magnetite is shown in Fig. 1. The peaks in the figure confirm evidently that the sample is magnetite. After washing the precipitate by magnetic decantation and by heating up to 80 °C, 0.4 ml of oleic acid and 1.1 g of sodium oleate were added to the nanoparticles suspension to prevent agglomeration. This mixture was stirred and heated at 70–80 °C during 30 min. Agglomerates were removed by centrifugation. Figure 2 presents the formation of particles with interdigitated bilayer. The stability of the ferrofluid was observed in time. The ferrofluid is found to be stable for more than 1 year.

## Measurements, results, and discussion

### Magnetic properties and granulometric analysis

Magnetic studies provide information on the concentration of magnetic material and nanoparticle-core size distribution (PSD). The mean magnetic diameter and its standard deviation are needed to compare ultrasonic data measured in different magnetic field strengths with the theoretical predictions.

Magnetic measurements of the sample studied were carried out with the aid of The Quantum Design MPMS 5XL Superconducting Quantum Interference Device (SQUID) Magnetometer which detects very small variations in magnetic flux and measures magnetic moment of the sample. From this, the magnetization and magnetic susceptibility can be determined. Data can be collected between *H* = 0 and 5 T in broad temperature range. The maximum sensitivity of the instrument is in the range of 10^−9^ emu. Samples are typically 20–40 mg but materials of strong magnetic properties can be measured with less material.

**Fig. 3:**
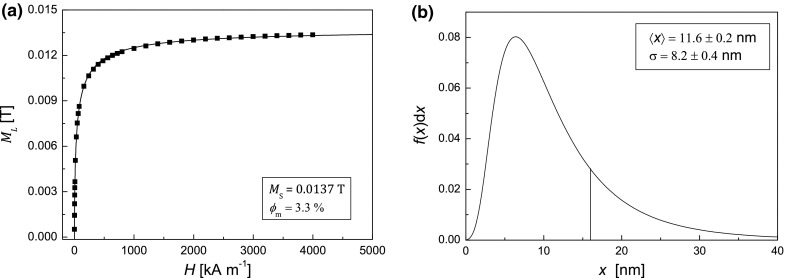
**a** Magnetization curve *M*(*H*) for studied samples obtained from SQUID data and **b** particle magnetic core size distributions

The results of SQUID measurements of the magnetization curve in a room temperature are shown in Fig. 3a. In order to determine saturation magnetization, *M*_s_, and to extract the PSD from magnetization curve the model employing the superposition of Langevin functions related to the different fractions of magnetic nanoparticles was used (Pshenichnikov et al. 1996; Rasa 2000):

1 $$ M_{\text{L}} = M_{\text{s}}\int\limits_{0}^\infty {L(\xi)f(x){\text{d}}x,} $$

where $L(\xi ) = \coth \xi - 1/\xi$ is the Langevin function, $\xi = \mu_{0} mH/k_{\text{B}} T$ is the Langevin parameter, *H* is the magnetic field strength, *k*_B_ is the Boltzmann constant, and *T* is the absolute temperature. The magnetic moment *m* = *M*_b_ *V* of the particle is proportional to its volume, *V*, and the bulk magnetization of the magnetic material *M*_b_. For the description of the dispersion of the particle size, the preferable choice is to use lognormal distribution (Pshenichnikov et al. 1996; Rasa 2000)

2 $$ f(x) = \frac{1}{{2S\sqrt {2\pi } }}\exp \left[ { - \frac{{\ln^{2} \left( {x/D_{0} } \right)}}{{2S^{2} }}} \right], $$

where *D*_0_ and *S* are the distribution parameters determined from the magnetization curve. Moments of arbitrary order *p* and standard deviation $\sigma$ can be calculated from the equations:

3 $$ {\text{x}}^{\text{p}} = D_{0}^{\text{p}} \exp \left( {\frac{{p^{2} S^{2} }}{2}} \right), $$

4 $$ \sigma = \sqrt {\left\langle x \right\rangle^{2} - \left\langle x \right\rangle^{2} } . $$

From the magnetization curve shown in Fig. 3a, the saturation magnetization *M*_s_ = 0.0137 ± 0.0001 T and parameters of lognormal distribution for the sample studied *D*_0_ = 9.47 ± 0.07 nm and *S* = 0.67 ± 0.02 were determined. Since a volume concentration *ϕ*_m_ of magnetic particles made of material with bulk magnetization *M*_b_ has a saturation magnetization *M*_s_ = *ϕ*_m_ *M*_b_, the volume concentration of the sample studied is *ϕ*_m_ = 3.3 % assuming bulk magnetization of magnetite to be *M*_b_ = 334 kA/m (Józefczak et al. 2012). Figure 3b shows particle size distribution function $f(x){\text{d}}x$ expressed as the probability of the particle size falling into the range of $[x,x + {\text{d}}x]$. The mean diameter of a magnetic nanoparticle and its standard deviation calculated from the Eqs. (3) and (4), respectively, are equal to $\left\langle x \right\rangle = 11.6 \pm 0.2$ nm and $\sigma = 8.2 \pm 0.4\,{\text{nm}}$.

The magnetic properties of magnetic nanoparticles, however, are reduced due to the presence of a magnetically inactive surface layer in which the atoms of a ferromagnetic material make no contribution to its total magnetic moment (Pshenichnikov et al. 1996). This effect influences the volume concentration of magnetic particles as well as their mean diameter determined by the analysis of magnetization curve. Assuming *δ*_m_ = 1 nm (Pshenichnikov et al. 1996; Rasa 2000) to be the thickness of a nonmagnetic surface layer, the true volume concentration calculated from the equation

5 $$ \phi_{\text{M}} = \phi_{\text{m}} \frac{{\left( {\left\langle x \right\rangle + \delta_{\text{m}} } \right)^{3} }}{{\left\langle x \right\rangle^{3} }} $$

is equal to 0.04. The true mean diameter $\left\langle x \right\rangle_{\text{M}}$ is, in turn, bigger than the mean diameter of the particle magnetic kernel obtained from the analysis of magnetization curve by magnitude 2*δ*_m_ and equals to 13.6 nm.

The coupling constant for the sample studied is $\lambda = 1.18.$ It means that, theoretically, magnetic interaction between the particles is too weak to provide their agglomeration into any aggregates. However, in polydisperse systems large particles with diameters larger than 16–18 nm corresponding to the tails of the particle size distribution are able to condense into cluster structures (Zubarev and Iskakova 2006). Given the particle size distribution function $f(x)$ one can calculate volume concentration of the larger particles ($d > 16\,{\text{nm}}$) from the following relation:

6 $${\phi _{16}} = {\phi _{\text{M}}}\int\limits_{16}^\infty  {f(x){\text{d}}x.} $$

For the magnetic nanofluid studied *ϕ*_16_ = 0.65 %. In rough estimation the effective coupling constant for the magnetic fluid studied, evaluated in accordance with the method given in (Wang and Holm 2003; Li and Li 2012), would be of 1.5. In a such bi-disperse model based on a mixture of ‘‘large’’ and ‘‘small’’ particles, which can be regarded as a system consisting of stronger and weaker interacting magnetic particles with different moments, the large particles are able to form aggregates, which would influence the ultrasonic behavior of the magnetic fluid (Taketomi 1986; Rozynek et al. 2011).

### Electron microscopy study

**Fig. 4:**
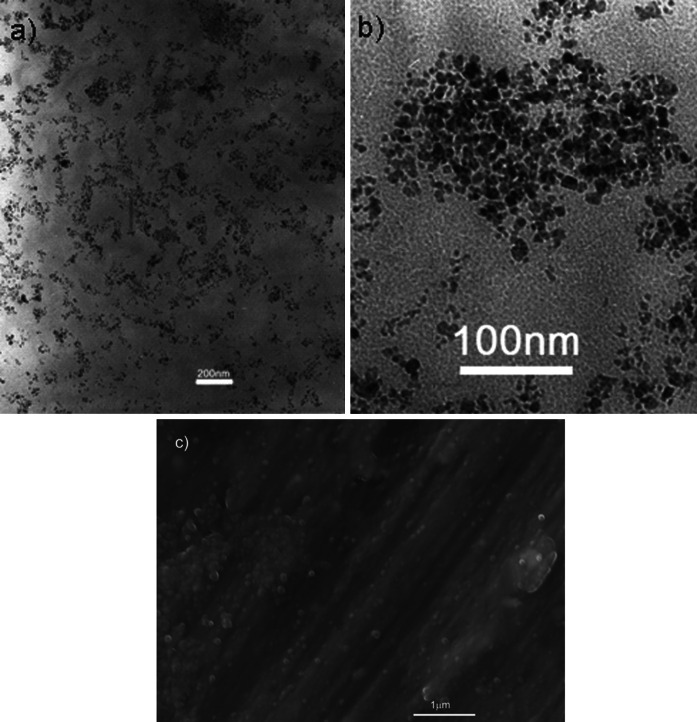
**a**, **b** TEM, and **c** SEM micrographs of synthesized Fe_3_O_4_ nanoparticles

**Fig. 5:**
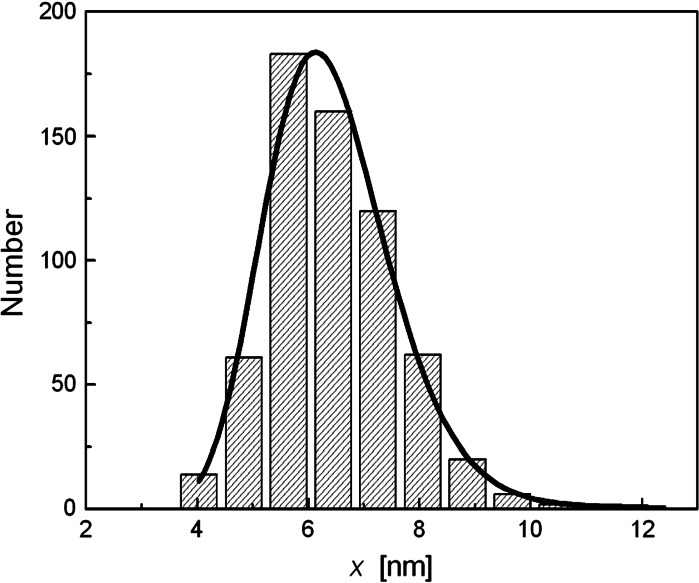
Particle magnetic core size distributions calculated from TEM images

The morphology of the synthesized particles was observed using transmission electron microscope TEM and scanning electron microscopy (SEM). Figure 4 shows the TEM and SEM images obtained from the magnetite nanoparticles, from which the size of magnetic core can also be extracted (Litvin et al. 2012; Litvin and Minaev 2013). The particle size analysis was carried out and its result, in the form of the particle size histogram, is shown in Fig. 5. The solid line represents the best-fit of lognormal distribution to the obtained histogram. The mean diameter of a magnetic nanoparticle and its standard deviation is equal to $\left\langle x \right\rangle = 6.23 \pm 0.07$ and $\sigma = 2.72 \pm 0.03$ nm. The smaller values of mean particle diameter and standard deviation observed from TEM data in comparison with those obtained from magnetic measurements may be due to the fact that the sample used for TEM experiment was very diluted in comparison with sample used in magnetic measurements. This results in the absence of magnetic interaction between nanoparticles which leads to the formation of small agglomerates detected in the SQUID experiment. On the other hand, ultrasonic measurements were carried out on nondiluted sample, so the granulometric analysis based on magnetic data seems to be more appropriate for the description of ultrasonic results.

### Determination of hydrodynamic size of magnetic nanoparticles covered with surfactant layers

After adding surfactants to the magnetic particles suspension, the nanoparticle size is increased. The mean core-shell nanoparticle diameter $\left\langle x \right\rangle_{\text{h}}$, which is called ‘‘hydrodynamic diameter”, is greater than the size of magnetic core by a magnitude 2*δ*_h_ = 2(*δ*_o_ + *δ*_s_),  where *δ*_o_ denotes the thickness of the first protective surfactant layer (oleic acid) and *δ*_s_ the thickness of the second surfactant layer (sodium oleate).

The hydrodynamic size distribution of nanoparticles in suspension was determined by dynamic light scattering (DLS) and differential centrifugal sedimentation (DCS). DLS evaluates the intensity fluctuation of scattered light reflected from magnetic nanoparticles in suspension. The fluctuation is resulting from the “Brownian motion” that keeps the particles in steady movement. The particle size measurements by DLS were carried out using a Malvern Zetasizer NanoZS. DCS determines particle size by measuring the time required for the colloidal particles to settle in a density gradient in a disk centrifuge. The DC24000 UHR disk centrifuge (CPS Instruments, Inc.) was used to perform sedimentation-based size distribution measurements.

**Fig. 6:**
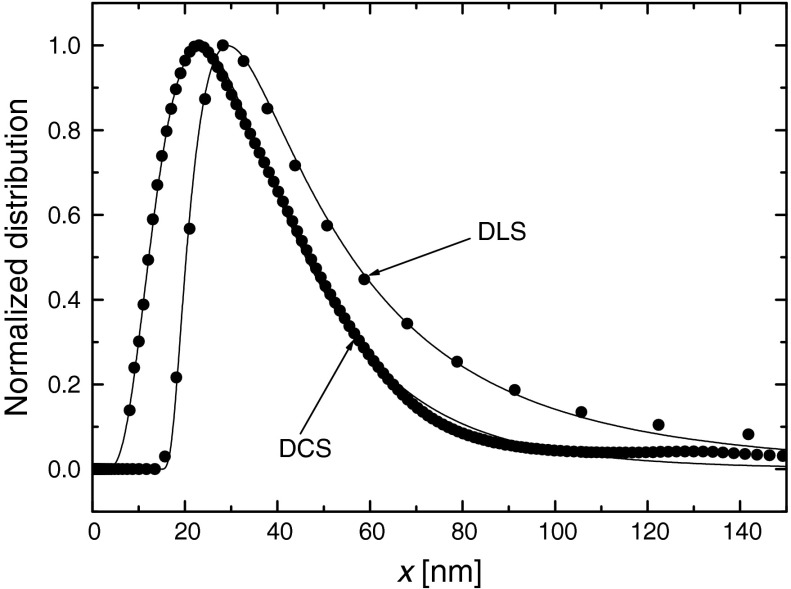
Particle hydrodynamic size volume distributions in the sample studied as measured using DLS and DCS methods. *Solid lines* were obtained by fitting the lognormal distributions to the experimental data

Figure 6 shows the results of normalized particle size distribution as measured using DCS and DLS methods. In order to determine the mean and standard deviation of the particle hydrodynamic sizes, the lognormal distribution was fitted to the experimental data. The results of fitting procedure are shown in Table 1. It is seen from the data in Table 1 that the thickness of both surfactant layers evaluated according to the DCS method would be of 7.05 nm and evaluated according to the DLS method would be of 11.45 nm. Since DCS has better sensitivity and resolution in the range of smaller particles, it was decided to use for further calculation the value of hydrodynamic diameter obtained from this method

**Table 1:** The values of the mean hydrodynamic diameter and its standard deviation as obtained using the DCS and DLS methods

Method	$\left\langle x \right\rangle_{\text{h}}$ (nm)	*σ*_h_ (nm)
DCS	27.7 ± 0.3	24.1 ± 0.3
DLS	36.5 ± 0.8	25.6 ± 1.1

### Rheological properties and density

In the analysis of sound propagation in colloid liquids within the framework of hydrodynamic theory, the emphasis is placed on the viscous and thermal phenomena in case of attenuation and elasticity and density in case of velocity. Ultrasound velocity measurement, together with precise density measurements, is a useful tool for determining the adiabatic compressibility.

**Fig. 7:**
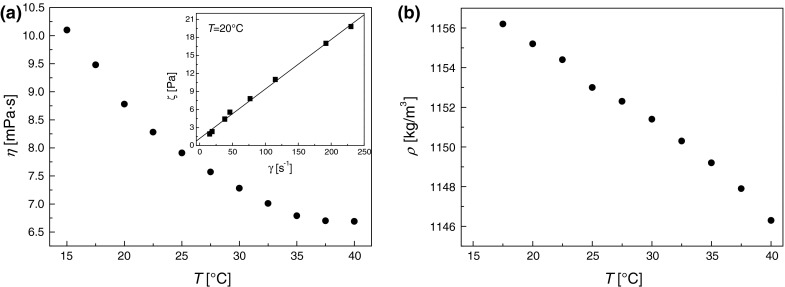
**a** Temperature dependence of the shear viscosity (the *inset* shows shear stress vs. shear rate flow curve) and **b** temperature dependence of the density of magnetic nanofluid

Rheological properties of magnetic liquid were measured using a rotational Digital Brookfield Rheometer DV II + in a cone-plate geometry, while the density was measured using a DMA-38 oscillating U-tube microprocessor densitometer from Anton Paar that operates on the method proposed by Kratky et al. (1973). In Fig. 7 the measured rheological properties and density of the magnetic nanofluid are plotted against temperature. The addition of coated nanoparticles to the carrier liquid—water in case of our sample—resulted in significantly increase of viscosity and density. The density decreases linearly with temperature. In the absence of the magnetic field, the viscosity decreases with temperature according to the Arrhenius equation. The inset in the Fig. 7a also shows flow curve, i.e., the shear stress as a function of the shear rate. The flow curves are linear within the shear rate range of measurement (20–250 s^−1^). It means that in the absence of magnetic field the magnetic fluid studied exhibits Newtonian behavior which is in agreement with other studies (Józefczak et al. 2013; Nowak and Odenbach 2013).

The magnetic fluid density *ρ* and the results of granulometric analysis described earlier can be used to determine magnetic particles volume concentration and also the volume concentration and the density of the particle aggregate formed by magnetic particle and stabilizing surfactant layer/layers. Providing that the properties of the surfactants on the particle surface are the same as for pure substances we can write (Vinogradov 2004)

7 $$ \phi_{\text{w}} + \phi_{\text{m}} + \phi_{\text{s}} = 1, $$

8 $$ \rho_{\text{w}} \phi_{\text{w}} + \rho_{\text{m}} \phi_{\text{m}} + \rho_{\text{s}} \phi_{\text{s}} = \rho , $$

where $\phi_{\text{w}} ,  \phi_{\text{m}} ,  \phi_{\text{s}}$ are the volume concentrations of water, magnetite, and surfactant, respectively. Densities of water, magnetite, and surfactant are, in turn, denoted by $\rho_{w} ,  \rho_{m} , \rho_{s}$, respectively. Assuming that the average aggregate density does not depend on the aggregate sizes but only on the densities of magnetite particles and surfactants forming stabilizing layers, the concentration $\phi$ and density *ρ*_a_ of the particle aggregate obey the following equations:

9 $$ \phi_{\text{m}} + \phi_{\text{s}} = \phi , $$

10 $$ \rho_{\text{m}} \phi_{\text{m}} + \rho_{\text{s}} \phi_{\text{s}} = \rho_{\text{a}} \phi . $$

Providing that all surfactant molecules are adsorbed on the surface of the magnetite particles the surfactant volume concentration is derived from the ratio of the volume of the surfactant bilayer to the volume of the magnetite particle (Vinogradov 2004)

11 $$ k = \frac{{\phi_{\text{s}} }}{{\phi_{\text{m}} }} = \frac{{6\delta_{\text{l}} }}{{{\text{x}}_{\text{m}} }} + \frac{{6\delta_{\text{l}} ({\text{x}}_{\text{m}} + 2\delta_{\text{l}} )^{2} }}{{{\text{x}}_{\text{m}}^{3} }}, $$

where *δ*_l_ is the thickness of each layer of surfactant. Since the densities of the sodium oleate and oleic acid differ only in about 5 %, *δ*_l_ is taken to be the half of *δ*_h_. On the basis of formulae (7)–(11), the volume concentration of magnetite and the density of aggregate can be determined from the expressions

12 $$ \phi_{\text{m}} = \frac{{\rho - \rho_{\text{w}} }}{{\rho_{\text{m}} - \rho_{\text{w}} - k(\rho_{\text{w}} - \rho_{\text{s}} )}} , $$

13 $$ \rho_{\text{a}} = \frac{{\rho_{\text{m}} \phi_{\text{m}} + \rho_{\text{s}} \phi_{\text{s}} }}{{\phi_{\text{m}} + \phi_{\text{s}} }}. $$

**Fig. 8:**
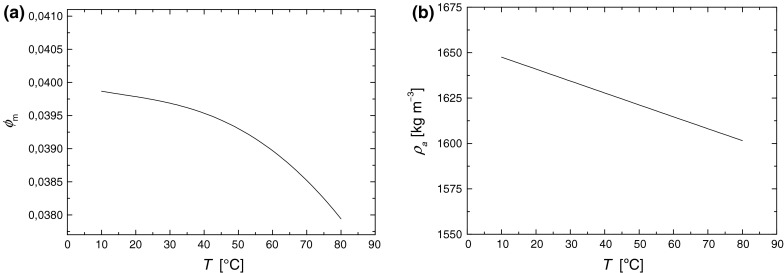
**a** The magnetite volume concentration, and **b** aggregate particle density as a function of temperature, calculated from the Eqs. (12) and (13)

The results of the calculation are shown in Fig. 8a, b. It should be noted that magnetite particles concentration for 20 °C coincides well with that obtained from magneto-granulometric analysis (Eq. 5). Particle aggregate density is much less than that of pure magnetite (5180 kg m^−3^ in 20 °C (Vinogradov 2004)). This means that particle aggregates are relatively soft, and the density contrast between the aggregates and surrounding fluid is rather small. In the calculation of $\phi ,  \phi_{\text{m}} , \;{\text{and}}\; \rho_{\text{a}}$ from the Eqs. (10), (12), and (13), the available literature density data for magnetite (Vinogradov 2004), oleic acid (Sharma et al. 2012), sodium oleate (Vinogradov 2004), and water (Kell 1975) were used.

### Ultrasonic properties in the absence of magnetic field

The velocity of ultrasonic wave in the absence of magnetic field was measured by means of a resonance method (Eggers and Kaatze 1996; Lautscham et al. 2000) using a ResoScan™System (Germany) apparatus. The ultrasonic velocity is determined from a series of resonance frequencies of the resonator cell recorded during initialization. Then, only a single resonance peak (chosen automatically) is tracked, and from the changes of resonance frequency of this peak, the ultrasonic velocity is evaluated. ResoScan™System permits the measurements of the ultrasonic velocity with the accuracy of ±0.01 m s^−1^ in two sample cells (0.200 ml capacity) in frequency range 7.3–8.4 MHz with temperature precision ±0.05 °C. Because of the high sensitivity from the multiple reflections of sound waves in resonators and because of the advantage of employing continuous wave signals, resonator techniques (sometimes named interferometers) are preferred for small volume sound velocity measurements.

**Fig. 9:**
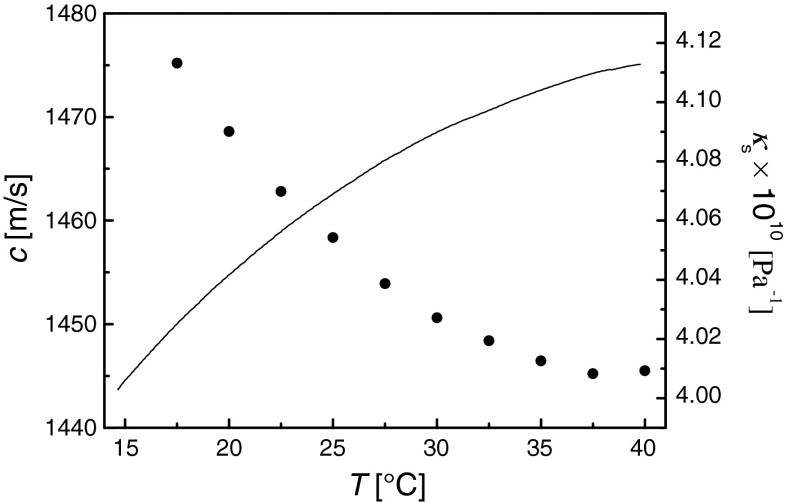
The measured ultrasonic wave velocity (*solid line*) and adiabatic compressibility (*dots*) as a function of temperature

Figure 9 shows the ultrasonic wave velocity as a function of temperature. It is seen from the figure that ultrasonic propagation velocity in magnetic suspension is smaller than that in carrier liquid, i.e., water ($v_{\text{w}} = 1509.2\,{\text{m}}\,{\text{s}}^{ - 1}$ for *T* = 30 °C (Marczak 1997)). According to the well-known Laplace equation *v* = (*ρκ*_*s*_)^−1/2^ (sometimes called Newton–Laplace or Wood equation), sound velocity of a fluid is related to its density *ρ* and adiabatic compressibility *κ*_s_, so it is possible to determine from speed of sound and density values, the elastic properties of a liquid characterized by the coefficient of adiabatic compressibility. The adiabatic compressibility of the suspension of nanoparticle coated by sodium oleate decreases with the increase in temperature (Fig. 9), similarly to that of suspension of nanoparticles coated Using PEG (Józefczak and Skumiel 2011).

As far as velocity of sound is considered, a suspension of one substance in another can be treated as a mixture of two materials, providing that the suspended particles are infinitesimally small compared to the wavelength of the sound, and that, accordingly, the scattering of the sound wave may be neglected. A magnetic fluid is such a mixture in which particle aggregates consisted of magnetic grains surrounded by a surfactant layer are suspended in a carrier liquid. Therefore, both the density and compressibility of the magnetic fluid can be written in the form (Povey 1997)

14 $$ \kappa_{\text{s}} = \phi \kappa_{\text{s}}^{\text{a}} + (1 - \phi )\kappa_{\text{s}}^{\text{w}} , \quad \rho = \phi \rho_{\text{a}} + \left( {1 - \phi } \right)\rho_{\text{w}} , $$

where *κ*_s_^a^ is the adiabatic compressibility of the particle aggregate, and *κ*_s_^w^ is the adiabatic compressibility of water. The compressibility and density obtained in such a way can be viewed as effective quantities characterizing liquid, and the speed of sound in magnetic fluid may be calculated form Laplace equation. This approach was originally proposed by Urick (1947) and later developed by Pinfield et al. (1995) who have shown that Urick equation can be written in the convenient following form

15 $$ \frac{1}{{v^{2} }} = \frac{1}{{v_{\text{w}}^{2} }}\left( {1 + \alpha \phi + \delta \phi^{2} } \right), $$

where *v* and *v*_w_ are the ultrasonic velocities in a magnetic fluid and water, respectively. The parameters *α* and *δ* are given by the following expressions

16 $$ \alpha = \frac{{\kappa_{\text{s}}^{\text{a}} - \kappa_{\text{s}}^{\text{w}} }}{{\kappa_{\text{s}}^{\text{w}} }} + \theta + \frac{{\rho_{\text{a}} - \rho_{\text{w}} }}{{\rho_{\text{w}} }}, $$

17 $$ \delta = \left( {\frac{{\kappa_{\text{s}}^{\text{a}} - \kappa_{\text{s}}^{\text{w}} }}{{\kappa_{\text{s}}^{\text{w}} }} + \theta } \right)\left( {\frac{{\rho_{\text{a}} - \rho_{\text{w}} }}{{\rho_{\text{w}} }}} \right) + \frac{{2(\rho_{\text{a}} - \rho_{\text{w}} )^{2} }}{{3\rho_{\text{w}}^{2} }}, $$

where

18 $$ \theta = \left( {\gamma_{\text{c}} - 1} \right)\frac{{\rho_{\text{a}} C_{\text{p}}^{\text{a}} }}{{\rho_{\text{w}} C_{\text{p}}^{\text{w}} }}\left( {\frac{{\beta_{\text{a}} \rho_{\text{w}} C_{\text{p}}^{\text{w}} }}{{\beta_{\text{w}} \rho_{\text{a}} C_{\text{p}}^{\text{a}} }} - 1} \right)^{2} . $$

In Eq. (18) $C_{\text{p}}^{\text{a}} ,  \beta_{\text{a}}$ are the specific heat and the volume thermal expansion coefficient, respectively, of particle aggregate and $C_{\text{p}}^{\text{w}} ,  \beta_{\text{w}}$ are those for water; *γ*_c_ is the ratio of the specific heats. The term *θ* was introduced to the Urick equation by Pinfield et al. (1995) to describe thermal effects which play very important role, especially in particulate media with small density contrast (e.g., emulsions) (Dukhin and Goetz 2002). This is the case in magnetic fluid since due to the softening effect of the surfactant layer the density contrast between particle aggregate and surrounding liquid is small. In suspension of solid particles in liquid dispersion medium thermal acoustic mode is a result of the coupling of pressure and temperature waves in particle and carrier liquid. The mode is generated to maintain continuity of temperature at the particle surface.

For the calculation of thermal expansion and heat capacity at constant pressure the following relations were used

19 $$ \beta_{\text{a}} = \frac{{\beta_{\text{m}} \phi_{\text{m}} + \beta_{\text{s}} \phi_{\text{s}} }}{{\phi_{\text{m}} + \phi_{\text{s}} }}, $$

20 $$ C_{\text{p}}^{\text{a}} = C_{\text{p}}^{\text{m}} x_{\text{m}} + C_{\text{p}}^{\text{s}} (1 - x_{\text{m}} ), $$

where

21 $$ x_{\text{m}} = \frac{{m_{\text{m}} }}{{m_{\text{m}} + m_{\text{s}} }} = \frac{{\rho_{\text{m}} }}{{\rho_{\text{m}} + k\rho_{\text{s}} }} $$

is the mass concentration of magnetite particles in the magnetite-surfactants aggregate. The literature data were used for thermal expansions and heat capacities of magnetite (Westrum and Grønvold 1969; Vinogradov 2004), oleic acid (Vinogradov 2004; Sharma et al. 2012), and water (Kell 1975; Lide 1992). The compressibility of water *κ*_s_^w^ was calculated from ultrasonic velocity (Marczak 1997) and density (Kell 1975) data (Table 2).

**Table 2:** The values of parameters $\alpha ,  \delta , \;{\text{and}}\; \theta$ obtained from the Eqs. (16)–(18) and particle aggregate adiabatic compressibility *κ*_s_^a^ determined on the basis of the best-fit of Urick equation (15) to the measured ultrasonic velocity data in the magnetic fluid studied

*T* (°C)	*α*	*δ*	*θ*	$\kappa_{\text{s}}^{\text{a}} \times 10^{10}$ (Pa^−1^)
15	0.106	−0.052	0.106	1.78
20	0.164	−0.032	0.098	1.92
25	0.196	−0.011	0.094	2.06
30	0.229	0.010	0.091	2.19
35	0.260	0.030	0.089	2.31
40	0.290	0.049	0.087	2.43

**Fig. 10:**
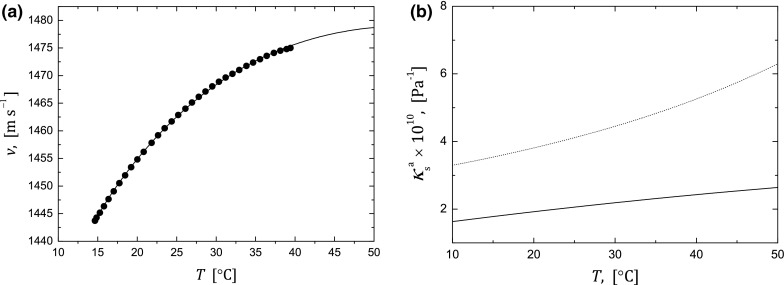
**a** The best-fit between experimental velocity data and Urick equation given by the formula (15), **b** The particle aggregate compressibility obtained from the best-fit between ultrasonic wave velocity and Urick equation (*solid line*). The *dotted line* shows particle aggregate adiabatic compressibility calculated form the Eq. (22)

The advantage of this approach to analysis of ultrasonic velocity data in particulate media lies in the fact that Urick Eq. (15) gives the adiabatic compressibility of the dispersed phase, that is *κ*_s_^a^. The solid line in Fig. 10a shows the temperature dependence of the ultrasonic velocity calculated from the Eqs. (16)–(21). From the condition of the best agreement between theory and experiment, the adiabatic compressibility of the dispersed phase (particle aggregates consisted of magnetite and surfactant layer) was determined and is shown in Fig. 10b. The dotted line in Fig. 10b denotes the adiabatic compressibility of the particle aggregate calculated as the volume fraction average of the compressibilities of the pure components:

22 $$ \kappa_{\text{s}}^{\text{a}} = \frac{{\kappa_{\text{s}}^{\text{m}} \phi_{\text{m}} + \kappa_{\text{s}}^{\text{s}} \phi_{\text{s}} }}{{\phi_{\text{m}} + \phi_{\text{s}} }}, $$

where $\kappa_{\text{s}}^{\text{m}} , \,\kappa_{\text{s}}^{\text{s}}$ is the compressibilities of magnetite (Reichmann and Jacobsen 2004) and oleic acid (Sharma et al. 2012), respectively. The discrepancy between adiabatic compressibility determined from ultrasonic velocity data and calculated from Eq. (22) may be caused by the state of the water in hydratation shell around magnetite particle.

### Anisotropy of ultrasonic wave propagation in magnetic fluid in the presence of the applied magnetic field

**Fig. 11:**
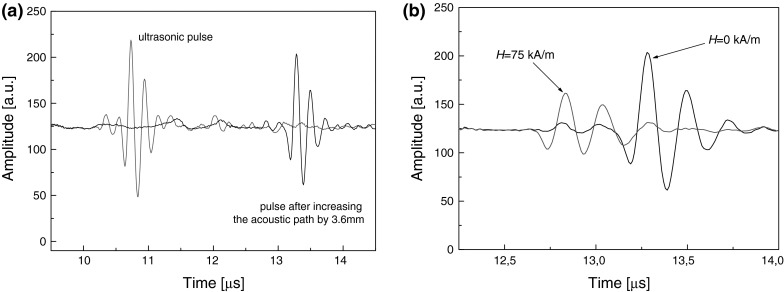
The time domain detected signals: **a** ultrasonic pulse after increasing the acoustic path by 3.6 mm; **b** measured pulse for magnetic fluid under the influence of magnetic field *H* = 75 kA m^−1^

**Fig. 12:**
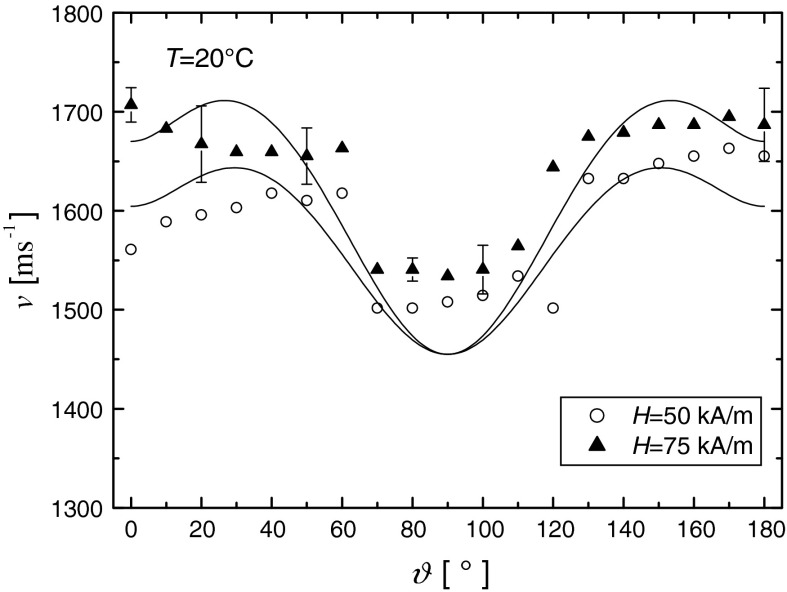
Anisotropy of the ultrasonic wave velocity in the magnetic nanofluid at different magnetic field values. The *dots* represent experimental values, and the *solid curves* show the theoretical predictions of the Eq. (24)

**Fig. 13:**
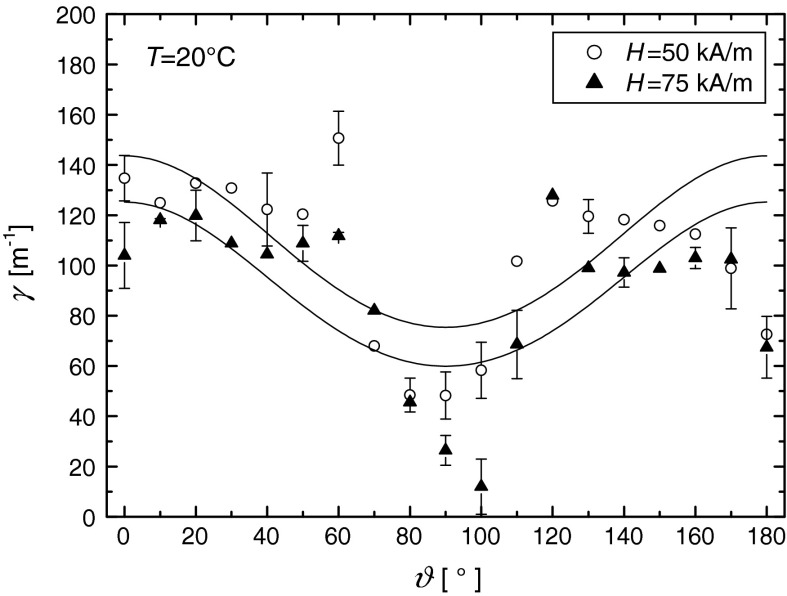
Anisotropy of the ultrasonic wave absorption in the magnetic nanofluid, at different values of the applied magnetic field values. The *solid lines* were obtained from the best-fit of the Eq. (26) to the experimental data

In the ultrasonic measurements under the influence of magnetic field, the pulse method was used. The piezoelectric transducer (Optel) with central frequency of 5 MHz operated in the pulse-echo mode was driven by Optel Pulser/Receiver Card 01/100 which provided a unipolar spike pulse with the amplitude of 360 V and fall time smaller than 20 ns. The received signal was sampled at a rate of 100 *M*_s_/s. The reflector in the measuring cell (made of brass) was moved a known distance with the help of the step motor (Charaziak et al. 2008). From the time of flight and the amplitude ratio of the ultrasonic pulses, the wave velocity and attenuation were determined. The analysis was performed on the received signals in time domain, and the attenuation coefficient *γ* was obtained from the following expression:

23 $$ \gamma = \frac{1}{\Delta l}\ln \frac{{A_{2} }}{{A_{1} }}, $$

where *A*_1_, *A*_2_ are the amplitudes of the signals transmitted through the sample, and *Δl* is the difference in the acoustic paths traveled by both signals (Fig. 11a). The propagation velocity of ultrasonic wave was determined from the relation *ν* = *l*/*t*, where *t* is the time in which pulse passes through the acoustic path *Δl*. The accuracy of the ultrasonic attenuation measurements described above, tested on a referenced fluid (castor oil), amounted to about ±2–5 % while the ultrasonic velocity was measured (in water) with the accuracy of ±0.5–1 %. However, in the magnetic fluid, the variation of the results between each series of measurements increased substantially the standard deviations of the mean values shown as an error bars in Figs. 12, 13.

In order to measure the effect of the magnetic field on the ultrasonic velocity and attenuation the measuring cell was placed between poles of an electromagnet. During anisotropy measurement the test cell remained stationary in the gap between pole pieces while the magnetic field was rotated by ten degrees each time. The magnetic field was measured with the accuracy of 0.5 % with a Resonance Technology RX21 type teslameter by placing the hall probe between pole pieces of the electromagnet when the magnetic fields was applied. Temperature in the sample cell was controlled by a bath thermostat (Polyscience model 9012) with the accuracy of ±0.05 °C.

Figure 11b shows the effect of magnetic field on the ultrasonic signal. The applying of the magnetic field influences both the velocity and attenuation of ultrasonic wave. The observed macroscopic anisotropy of the ultrasonic properties, that is the dependence of the ultrasonic wave velocity and attenuation on the direction of the wave with respect to the magnetic field, was similar to that in other magnetic fluids (Skumiel 2004; Józefczak and Skumiel 2006; Motozawa et al. 2008; Rozynek et al. 2011; Kúdelčík et al. 2013). There are two main mechanisms responsible for the ultrasonic anisotropy in magnetic fluids: magnetization relaxation and internal chain dynamics. Relaxation of the actual magnetization to the new equilibrium is accompanied with dissipation of ultrasonic wave energy, however, as was shown in (Müller et al. 2003) the contribution of this mechanism to the ultrasonic attenuation is negligible in MHz frequency range. On the other hand, this mechanism seems to be important in case of ultrasonic velocity (Ovchinnikov and Sokolov 2009, 2013). The second mechanism of sound attenuation is attributed to the internal chain dynamics and was introduced by Taketomi (1986) who assumed that compression or stretching of the chain-like aggregates aligned with field direction under the influence of the ultrasonic wave results in a magnetic restoring force leading to the forced oscillations of the aggregates. Taketomi’s model was further refined in the papers of Pleiner and Brand (1990) and Shliomis et al. (2008).

Ovchinnikov and Sokolov theory (Ovchinnikov and Sokolov 2009, 2013) considers the propagation velocity of acoustic waves in a magnetic fluid under the influence of external magnetic field. They assumed magnetic fluid to be ideal with no attenuation whatsoever and the model of frozen-in magnetization, i.e., the case where the magnetization relaxation time is infinitely large. As a result the applied magnetic field $H$ differs from the equilibrium magnetic field strength $H^{\text{eq}}$ in the fluid. Using the set of hydrodynamic equations for magnetic fluid and the Maxwell magnetostatic equations (Ovchinnikov and Sokolov 2009, 2013), they show that in a magnetic fluid with frozen-in magnetization, three hydrodynamic modes are present: the Alfvén waves, the fast magnetoacoustic wave, and the slow magnetoacoustic wave. In a typical scenario of ultrasonic experiment only the fast magnetoacoustic wave can be measured. Its velocity according to Ovchinnikov and Sokolov theory is given by (Ovchinnikov and Sokolov 2013)

24 $$ v = \left\{ {\frac{1}{2}\left( {v_{0}^{2} + v_{\text{A}}^{2} \left( {\frac{{\beta_{\parallel } }}{{\beta_{ \bot } }} + 1} \right) + \sqrt {\left( {v_{0} + v_{\text{A}}^{2} \left( {\frac{{\beta_{\parallel } }}{{\beta_{ \bot } }} - 1} \right)} \right)^{2} + 4v_{0}^{2} v_{\text{A}}^{2} \left( {1 - \frac{{\beta_{\parallel } }}{{\beta_{ \bot } }}} \right)\sin^{2} \vartheta } } \right)} \right\}^{1/2} , $$

where *v*_0_ is the ultrasonic velocity in the absence of a magnetic field, $v_{\text{A}} = m_{0} \sqrt {\beta_{ \bot } } \cos \vartheta$ is the phase velocity of the Alfvén-type wave, *m*_0_ = *M*_0_/*ρ* is the specific magnetization, $\beta_{\parallel }$ and $\beta_{ \bot }$ are the components of the diagonal tensor that determines the magnetoelastic properties of the magnetic fluid, and *ϑ* is the angle between the wave vector and the applied magnetic field. The specific magnetization of the magnetic fluid as a function of the external magnetic field was determined from the Langevin equation (1) using mean diameter of magnetic particles extracted from magnetization curve:

25 $$ m_{0} = \frac{{\phi_{\text{m}} M_{\text{b}} }}{\rho }L\left( \xi \right). $$

Figure 12 shows the experimental values of ultrasonic velocity as a function of the angle between wave vector and magnetic field direction. The ultrasonic velocity anisotropy expressed as $\Delta v = v\left( 0 \right) - v(90^\circ )$, that is the difference in velocity between the wave propagating parallel and perpendicular to the applied magnetic field, increases from 54 m s^−1^ for the magnetic field of 50 kA m^−1^ to 172 m s^−1^ for the magnetic field of 75 kA m^−1^.

The solid lines in Fig. 12 show predictions of Ovchinnikov and Sokolov theory (Ovchinnikov and Sokolov 2009, 2013) expressed in the Eq. (24). The theoretical lines, at least qualitatively, reproduce experimental velocity data for the values of diagonal magnetoelastic tensor $\beta_{\parallel }$ and $\beta_{ \bot }$ listed in Table 3. According to the theory (Ovchinnikov and Sokolov 2009, 2013), the shape of the experimental dependence of the ultrasonic velocity anisotropy expressed by $\Delta v(\vartheta ) = v\left( \vartheta \right) - v(90^\circ )$ allows one to determine the type of mechanism responsible for the ultrasonic velocity anisotropy. If the $\Delta v\left( \vartheta \right) > 0$, which is the case for the magnetic fluid studied in this article for the angles within 0°–90°, then the governing mechanism is magnetoelastic.

**Table 3:** The values of specific magnetization *m*_0_ calculated from the Eq. (25) and the components of the diagonal magnetoelastic tensor $\beta_{\parallel }$ and $\beta_{ \bot }$ determined from the best-fit of the Eq. (24) to the experimental ultrasonic velocity data

*H* (kGs)	*m*_0_ (Gs cm^3^ g^−1^)	$\beta_{\parallel } \times 10^{ - 7} \;({\text{g}}\,{\text{cm}}^{ - 3} )$	$\beta_{ \bot } \times 10^{ - 7} \;({\text{g}}\,{\text{cm}}^{ - 3} )$
0.6	13.8	2.4	7.5
1.0	14.7	3.1	8.5

Figure 13 shows the experimental values of ultrasonic attenuation as a function of the angle *ϑ* between the wave vector and the magnetic field direction. There is an approximate monotonic decrease of the ultrasonic attenuation with increasing *ϑ*, and the attenuation is maximal when the field is applied parallel to the direction of the sound propagation and minimal when the field is applied perpendicularly. The level of attenuation is about three times higher than that of water for 5 MHz.

The observed dependence of $\gamma_{ \dim }$ on *ϑ* is characteristic of short-chains aggregates that are formed as a result of the pair interparticle magnetic dipole–dipole interaction (Shliomis et al. 2008). Although the coupling constant for the magnetic fluid studied in this article is close to unity (*λ* = 1.18), which means that most nanoparticles are too small to join into chain-like structures, the relatively high content of bigger particles (larger than 16 nm) makes possible to form, at least, two-particles chains (dimers) which cause the ultrasonic attenuation due to the dimer formation to be comparable with viscous one. The evidence of such changes in magnetic fluid microstructure is the unusually high increase of ultrasonic attenuation with the applied magnetic field. The ultrasonic attenuation in the absence of the external magnetic field is of the order of 8 m^−1^ and rises about 15 times to 120 m^−1^ in the field of 50 kA m^−1^ applied parallel to the direction of the ultrasonic wave propagation. Also the level of ultrasonic attenuation anisotropy is of 50 % that means the attenuation of sound waves propagating perpendicular to the applied magnetic field decrease by half compared to that propagating parallel to the applied magnetic field.

According to Shliomis et al. (2008) the two-particle chains (dimers) oscillations in the ultrasonic field lead to the additional dissipation of ultrasonic wave energy with coefficient of absorption given by

26 $$ \gamma_{ \dim } = \frac{{\omega^{2} \eta \phi_{ \dim } }}{{2\rho v^{3} }}{\left\langle {\overline {r}} \right\rangle }^{2} \frac{{\omega^{2} \tau^{2} }}{{1 + \omega^{2} \tau^{2} }}F\left( \vartheta \right), $$

where $v$ is the velocity of the ultrasonic wave propagating with angular frequency *ω*, *τ* is the relaxation time of dimer oscillations scaled by the Brownian diffusion time *τ*_B_ = 3*ηV*_h_/*k*_B_ *T* for a single particle, *ρ* is the density, and *η* the shear viscosity of the magnetic liquid, respectively, $\phi_{ \dim }$ is the volume fraction of the dimers, and $\overline{r}$ is the ratio of the mean distance between magnetic particles to their average diameter. The anisotropy function $F(\vartheta )$ can be expressed by a field-induced nematic order parameter $S(\lambda ,\xi ) = \frac{1}{2}\left( {3\left\langle {\cos^{2} \alpha } \right\rangle - 1} \right)$, *α* being the angle between the dimer axis and the applied magnetic field, through the relation

27 $$ F\left( \vartheta \right) = \left[ {1 + S(3\cos^{2} \vartheta - 1)} \right]^{2} . $$

The solid lines in Fig. 13 were obtained by fitting the Eqs. (26) and (27) following from the Shliomis et al. (2008) theory to the experimental data. The values of nematic order parameters obtained from fitting procedure are *S* = 0.11 for the magnetic field of 50 kA m^−1^ and *S* = 0.13 for the field of 75 kA m^−1^. They are comparable with the values of 0.15–0.18 resulting from the theory of Shliomis, Mond, and Morozov (Fig. 2 in Shliomis et al. 2008).

## Conclusions

Ultrasonic wave propagation in the magnetic fluid consisted of magnetite nanoparticles suspended in water in the absence as well as in the presence of the applied magnetic field has been studied. The granulometric analysis based on the magnetization, DLS and DCS measurements was performed. It showed that the magnetic fluid studied in this article is polydispersed, and the mean particle hydrodynamic size is twice as much as the mean size of the magnetite grains due to the presence of double surfactant layer surrounding each particle. Using modified Urick theory as well as the measured data of ultrasonic velocity in the absence of applied magnetic field, the adiabatic compressibility of the particle aggregate consisted of the magnetite core and surfactant layers was determined. In the external magnetic field, the studied magnetic fluid showed an ultrasonic anisotropy, i.e., the dependence of the velocity and attenuation on the angle between the wave vector and the direction of the magnetic field. It was showed that the ultrasonic velocity anisotropy was due to the relaxation of magnetization whereas the anisotropy of the attenuation was caused by the short, mainly two-particle, chains aligned with the magnetic field.
